# 

**Published:** 1908-05

**Authors:** 


PUBLISHED MONTHLY. SUBSCRIPTION $1.00

### ATLANTA JOURNAL-RECORD OF MEDICINE

Successor to { **Atlanta Medical and Surgical Journal, Established 1855.** / **and Southern Medical Record, Established 1870.** } 53d Year

Vol. X. ATLANTA, GA., MAY, 1908. No. 2

